# Adult autoimmune enteropathy presenting initially with acquired Acrodermatitis Enteropathica: a case report

**DOI:** 10.1186/s12895-017-0059-4

**Published:** 2017-05-18

**Authors:** Erina Lie, Sarah Sung, Steven Hoseong Yang

**Affiliations:** 1 Department of Dermatology, Johns Hopkins University School of Medicine, 200 North Wolfe Street, Unit 2106, Baltimore, MD 21287 USA; 2 Department of Dermatology, Johns Hopkins University School of Medicine, 1550 Orleans Street, Koch CRB II, Unit 206, Baltimore, MD 21231 USA; 3 Department of Dermatology, Johns Hopkins University School of Medicine, 1550 Orleans Street, Koch CRB II, Unit 206, Baltimore, MD 21231 USA

**Keywords:** Acrodermatitis enteropathica, Zinc deficiency, Dermatitis, Autoimmune enteropathy, Malnutrition, Case report

## Abstract

**Background:**

Acrodermatitis enteropathica (AE) is a rare dermatitis secondary to zinc deficiency most commonly seen as an inherited disease in infants. In the last decade, increased number of reports have been published on the acquired form that presents in adulthood. Unlike its inherited counterpart, acquired AE (AAE) is often secondary to underlying pathologic or iatrogenic etiologies that interfere with nutritional absorption, such as inflammatory bowel disease or alcoholism. Various gastrointestinal pathologies have been associated with AAE, but there is currently no report on its association with adult autoimmune enteropathy (AIE), a rare gastrointestinal disorder commonly seen in infants, with limited cases reported in adults. Here we present a case in which AAE was the initial clinical manifestation in an adult patient subsequently diagnosed with AIE.

**Case presentation:**

A 41-year-old African American female presented to our emergency department at the Johns Hopkins Hospital with several months of progressively worsening dermatitis in the legs and acral regions, along with worsening symptoms of diarrhea, alopecia, poor oral intake, lethargy, hematochezia, peripheral edema, and weight loss. Our dermatology team was consulted given a presentation of exquisitely tender, erythematous, and diffusely desquamating skin lesions in the setting of two prior outside hospitalizations in the last 3 months with the same dermatitis that was refractory to topical and oral corticosteroids. Low serum zinc level and positive response to zinc supplementation confirmed the diagnosis of AAE. However, persistent hypovitaminosis and mineral deficiency despite aggressive nutritional supplementation prompted further investigation for an underlying malabsorption etiology. Jejunal biopsy and associated autoantibodies confirmed a diagnosis of adult AIE.

**Conclusion:**

This case highlights the fact that adult AIE can present initially with clinical findings of AE. While proper zinc supplementation can resolve the latter, recognizing this association can trigger earlier diagnosis, minimize unnecessary tests, and establish earlier intervention to improve quality of life and prevent recurrence of AAE. The case also highlights the importance of collaboration between general and subspecialist physicians in identifying a primary etiology to a secondary clinical presentation. This report can be beneficial to general internists and emergency physicians, as much as it can be to dermatologists, rheumatologists, and gastroenterologists.

## Background

Acrodermatitis enteropathica (AE) is a condition caused by zinc deficiency that classically presents with periorificial and acral papulosquamous eruptions accompanied by diarrhea and alopecia [1]. The inherited form, a rare autosomal recessive trait due to mutations in the gene SCL39A4 on chromosome 8q24.3 [2], is more common and typically seen in infants. However, in the last decade, we have witnessed an increased number of reports on acquired AE affecting adults and the elderlies particularly in association with anorexia nervosa, alcoholism, bariatric surgery, total parenteral nutrition, nephropathy, inflammatory bowel disease (IBD), blind loop syndrome, and celiac disease [3–7]. Here we present a novel case of acquired AE as an initial presentation of autoimmune enteropathy (AIE), itself a rare gastrointestinal disorder characterized by refractory diarrhea and malnutrition that affects less than 1/100,000 infants, with limited case reports in adults [8, 9].

## Case presentation

**Fig. 1:**
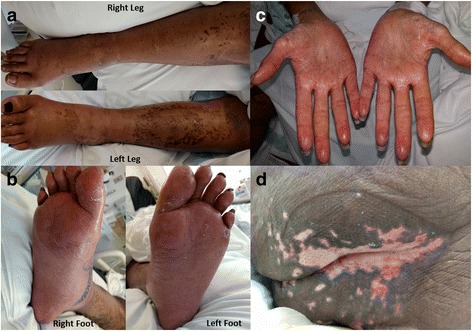
Acquired acrodermatitis enteropathica – Day 2 of admission. Legend: Desquamative erythematous patches with edema involving bilateral lower extremities (**a**) and plantar feet (**b**). Erythema with subtle desquamation on bilateral palmar hands (**c**). Ulceration and satellite erosions in the lumbosacral, perianal, and perineal region (**d**)

A 41-year-old African American female presented with a 4-month history of progressive, exquisitely tender, non-pruritic leg and acral dermatitis, diarrhea, alopecia, poor oral intake, lethargy, hematochezia, lower extremity edema, and 22lbs of unintentional weight loss. Physical examination revealed erythema and desquamation along the extremities (Fig. 1a), plantar feet (Fig. 1b), and palmar hands (Fig. 1c), paronychia, angular cheilitis with lip fissure, glossitis, ulcerations and satellite erosions in the lumbosacral, perianal, and perineal regions (Fig. 1d), and diffuse alopecia of the scalp.

Her symptoms began initially with dysgeusia, lower extremity edema, along with erythematous cutaneous eruption and desquamation of the lower extremities and acral region that was refractory to topical triamcinolone 0.1% ointment. By the second month, her symptoms persisted with additional blurring of vision, xerostomia, diarrhea, hematochezia, anorexia, and thrombocytopenia that necessitated a 22-day hospitalization. She was diagnosed with immune thrombocytopenic purpura (ITP) and non-alcoholic steatohepatitis, confirmed by positive anti-platelet antibody and liver biopsy, respectively. She was started on prednisone 60 mg and discharged with a 4-week taper.

By the third month, specifically a day after discontinuing prednisone, she noted recurring edema in her lower extremities that progressed rapidly to painful desquamative and vesiculobullous lesions resulting in a second hospitalization. Skin biopsy revealed mild spongiotic dermatitis with alternating hyperparakeratosis and papillary dermal edema without evidence of vasculitis, systemic lupus erythematous, or autoimmune bullous disease on immunofluorescence. She was diagnosed with an eczematous dermatitis and discharged with another 4-week prednisone taper. During this hospitalization, she also developed sacral pressure ulcer. Two weeks after this last hospitalization, persistent edema, a non-healing sacral ulcer, and worsening desquamative plaques eventually brought her to our institution for the first time with the presentation described above.

**Fig. 2:**
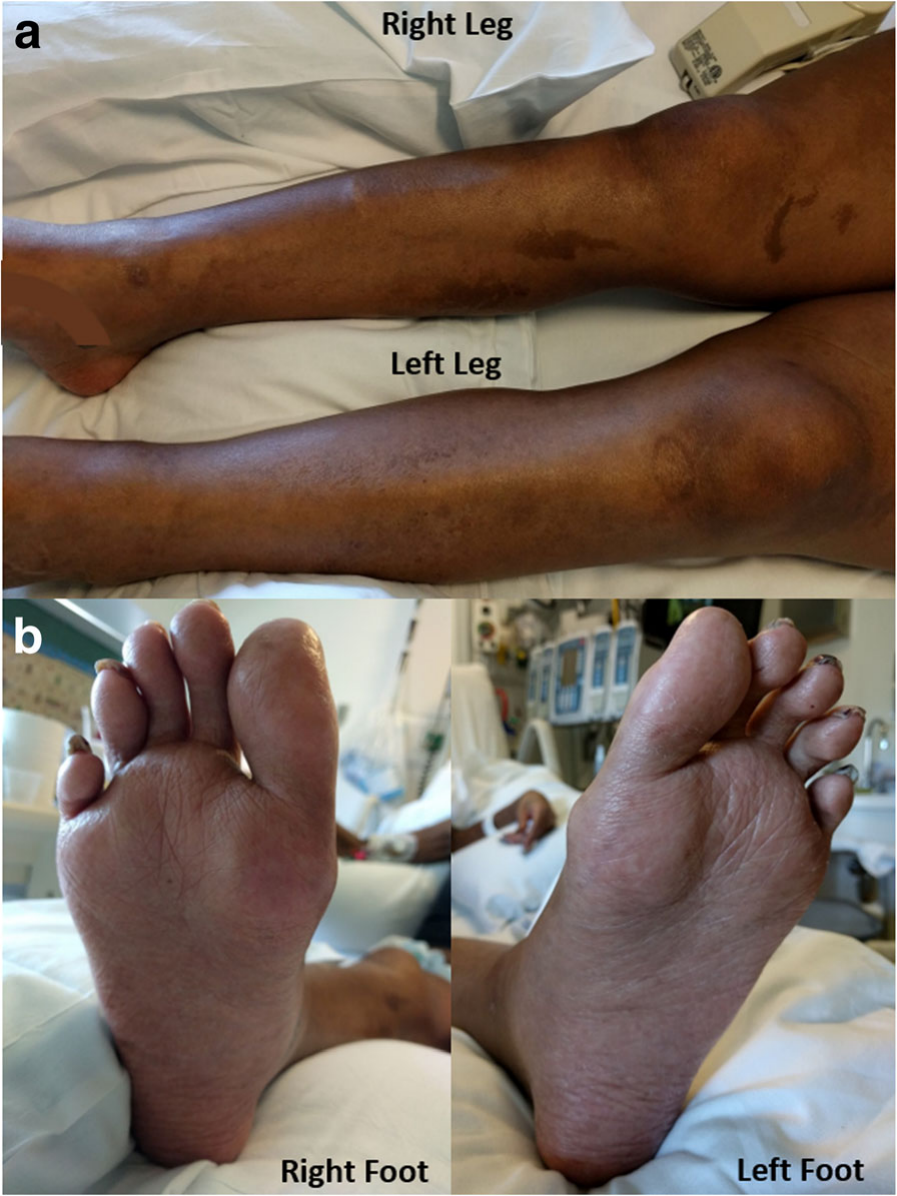
Acquired acrodermatitis enteropathica – Day 20 of admission. Legend: Resolution of edema, erythema, and desquamation in the bilateral lower extremities (**a**) and plantar feet (**b**)

Repeat skin biopsy of the right medial malleolus demonstrated an unremarkable epidermis, slightly ectatic superficial dermal vessels with surrounding focal rare lymphocytes and rare extravasated red blood cells, without evidence of erythema multiforme or Stevens-Johnson Syndrome/Toxic Epidermal Necrolysis (SJS/TEN). However, her zinc level was found to be 30 μg/dL (normal: 60-130 μg/dL), which improved after 20-days of intravenous supplementation of zinc sulphate 220 mg thrice daily (Table 1). Clinically, her cutaneous findings and gastrointestinal function also showed marked improvements (Fig. 2a-b). Her dermatologic findings and responsiveness to zinc supplement confirmed the diagnosis of acquired AE.

**Table 1:** Serum nutrient levels (values at initial presentation and prior to discharge) and nutrient repletion regimens during the 20 days of hospitalization

Serum Level	Initial Value	Value after 15–20 Days	Reference Range	Repletion
Pre-albumin^ab^	7 mg/dL	7 mg/dL	18–38 mg/dL	-
Albumin^ab^	1.5 g/dL	2.3 g/dL	3.5–5.3 g/dL	-
Ionized Calcium^a^	1.07 mmol/L	1.20 mmol/L	1.13–1.32 mmol/L	-
Copper^ab^	51 μg/dL	67 μg/dL	70–175 μg/dL	2 mg PO daily
Iron^ab^	48 μg/dL	44 μg/dL	50–170 μg/dL	325 mg PO with meals
Magnesium	2.0 mg/dL	1.8 mg/dL	1.6–2.4 mg/dL	-
Phosphorus^a^	2.5 mg/dL	3.0 mg/dL	2.7–4.5 mg/dL	-
Selenium^ab^	44 μg/dL	48 μg/dL	63–160 μg/dL	50mcg PO daily
Zinc^ab^	30 μg/dL	52 μg/dL	60–130 μg/dL	220 mg PO TID^d^
Vit A^ab^	11 μg/dL	14 μg/dL	38–98 μg/dL	200,000 IU PO ×3
Vit B1	109 nmol/L	-	78–185 nmol/L	100 mg PO daily
Vit B3/Niacin	<20 ng/mL	-	Variable	100 mg PO qhs
Vit B6^ab^	<2.0 ng/mL	-	2.1–21.7 ng/mL	100 mg PO daily
Vit B9/Folate	1743 ng/dL	1578 ng/dL	>498 ng/dL	-
Vit B12	1161 pg/mL	1592 pg/mL	211–946 pg/mL	-
Vit D total^ab^	18 ng/mL	8 ng/mL	30–100 ng/mL	Vit D_2_: 50,000 IU PO q7d CaCO_3_: 1300 mg PO TID
Vit E – **α** ^abc^	1.1 mg/L	2.5 mg/L	5.7–19.9 mg/L	100 mg PO daily
Vit E – **β** ^c^	0.4 mg/L	1.0 mg/L	≤4.3 mg/L	100 mg PO daily
Vit K	84 pg/mL	-	80–1160 pg/mL	-

^a^Initial value is below normal

^b^Latest value prior to discharge is below normal

^c^α = alpha tocopherol; β = beta tocopherol

^d^200 mg zinc sulphate tablet contains 50 mg of elemental zinc

Notably, she had severe hypovitaminosis and mineral deficiencies that improved only minimally despite repletion (Table 1). Given a newly-diagnosed ITP, hematochezia, sicca syndrome, and persistently low pre-albumin level, she underwent an extensive additional work-up to investigate the possibility of an underlying etiology contributing to her nutritional deficiency. Her diagnostic work-up included broad titer analyses (Tables 2 and 3), blood/urine/stool analyses, esophagogastroduodenoscopy with biopsy, and colonoscopy with biopsy. Negative biopsy findings and associated autoantibody, viral, fungal, and bacterial titers ruled out the more common autoimmune and infectious causes of malabsorption, such as IBD and celiac disease (Table 3). Our patient was eventually diagnosed with AIE based on jejunal biopsy findings of mild increase in intraepithelial lymphocytes, crypt apoptosis, reactive epithelial changes, and mild mononuclear and neutrophil expansion of the lamina propria, supported by positive anti-gastric parietal cell and anti-smooth muscle autoantibodies. Other reported associations with AIE, including sicca syndrome, gastritis, nephrotic syndrome, autoimmune hepatitis, and chronic pancreatitis, were also present in this patient, further supporting the diagnosis. She was discharged after 20 days with central parenteral nutrition (CPN), prednisone 15 mg daily, and close outpatient follow-up with markedly improved dermatologic findings. Follow-up visit at our dermatology clinic 15 weeks later was unremarkable with no recurrence of cutaneous findings (Table 4). At the time of manuscript submission, patient has remained stable without further hospitalization nor recurrence of symptoms. She continued to require CPN and oral supplements with weekly nutritionist monitoring and periodic gastroenterology follow-up.

**Table 2:** Abnormal titre results for autoantibodies, viruses, fungi, and bacterial toxins

Titre Names	Results	Reference Range
Abnormal results:		
Anti-Ro (SSA) Ab	Moderate Positive	Negative
Gastric parietal cell Ab	55.5 units	≤20 units = Negative
Smooth muscle Ab	Positive	Negative
C3, serum	71 mg/dL	79–251 mg/dL
CMV viral load	2640 IU/mL	<137 IU/mL

*Ab* = antibody

*CMV* = Cytomegalovirus

**Table 3:** Titre results within normal range for autoantibodies, viruses, fungi, and bacterial toxins

Titre Names		
Alpha-1 anti-trypsin, stool, 24-h	Anti-Jo-1 Ab	Direct anti-globulin test
p-ANCA	Anti-La (SSB) Ab	Glomerular basement membrane Ab
c-ANCA	Anti-nuclear Ab (ANA)	Islet cell Ab
Anti-cardiolipin Ab, IgG	Anti-Scl70 Ab	Liver-kidney microsomal Ab
Anti-cardiolipin Ab, IgM	Anti-Smith Ab	Mitochondrial Ab
Anti-cardiolipin Ab, IgA	Anti-URP Ab	Rheumatoid factor
Anti-dsDNA Ab	Beta-2 glycoprotein Ab, IgG	Thyroglobulin Ab
Anti-enterocyte Ab, IgG	Beta-2 glycoprotein Ab, IgM	Thyroid peroxidase microsomal Ab
Anti-enterocyte Ab, IgM	Beta-2 glycoprotein Ab, IgA	Tissue transglutaminase, IgA
Anti-enterocyte Ab, IgA	Cyclic citrul peptide Ab	TSH receptor Ab
C4, serum	EBV viral load	*C.difficile* toxin B gene NAT
CH50, serum	(1–3)-Beta-D-Glucan	Diphtheria antitoxin Ab
	Galactomannan, serum	

*Ab* = antibody

*p-ANCA* = perinuclear anti-neutrophilic cytoplasmic antibody

*c-ANCA* = cytoplasmic anti-neutrophilic cytoplasmic antibody

*ANA* = Antinuclear Antibody

*EBV* = Epstein-Barr Virus

*TSH* = Thyroid-Stimulating Hormone

**Table 4:** Case report timeline

Chronology	Timeline Description
T_0_–4 months	Clinical presentation: dysgeusia, lower extremity edema, and cutaneous eruption and erythema of the lower extremities and acral region with desquamation Management: refractory to topical triamcinolone 0.1% ointment
T_0_–3 months	Clinical presentation: symptoms persisted with additional blurring of vision, xerostomia, diarrhea, hematochezia, anorexia, and thrombocytopenia Diagnosis: immune thrombocytopenic purpura (ITP) and non-alcoholic steatohepatitis Management: 22-day hospitalization, prednisone 60 mg and discharged with a 4-week taper
T_0_–2 months	Clinical presentation: recurring edema in the lower extremities progressing rapidly to painful desquamative and vesiculobullous lesions Diagnosis: eczematous dermatitis Diagnostic tests: skin biopsy Management: second hospitalization, discharged with another 4-week prednisone taper Comments: developed sacral pressure ulcer
T_0_	Clinical presentation: persistent edema, non-healing sacral ulcer, worsening desquamative plaques Diagnosis: acquired acrodermatitis enteropathica and severe nutrition deficiency Diagnostic tests: skin biopsy of the right medial malleolus, broad titre analyses, esophagogastroduodenoscopy and colonoscopy with biopsy Management: broad nutrition repletion
T_0_ + 3 weeks	Clinical presentation: marked improvements of cutaneous findings and gastrointestinal function Diagnosis: adult autoimmune enteropathy Management: discharged with central parenteral nutrition (CPN), prednisone 15 mg daily, and close outpatient follow-up
T_0_ + 4 months	Clinical presentation: outpatient follow-up with unremarkable cutaneous findings Management: continue CPN and oral supplements, close outpatient follow-up with gastroenterology and nutrition

## Discussion

Zinc is an essential mineral that plays crucial roles in metabolism, development, tissue repair, and cell proliferation, including proper maturation of basal keratinocytes [1]. Zinc deficiency, manifested in AE, can be acquired through decreased intake (e.g. vegetarianism, alcoholism), increased demand (e.g. pregnancy), intestinal malabsorption (e.g. IBD, gastric bypass), increased urinary loss (e.g. diuretics), or state of hypoalbuminemia since zinc binds albumin in the circulation (e.g. liver damage) [1]. Aside from the triad of dermatitis, diarrhea, and alopecia, symptoms of AE can also include angular cheilitis followed by paronychia, glossitis, ophthalmologic disturbances, poor wound healing, anemia, dysgeusia, dysosmia, and profound lethargy [1, 10]. Differential diagnoses include necrolytic migratory erythema, SJS/TEN, blistering diseases, epidermolysis bullosa, and pellagra [6, 10]. Histopathologic findings are typically indistinguishable from other forms of malnutrition dermatitis. Pathognomonic feature of fully-developed necrolysis has been reported, which involves cytoplasmic pallor, vacuolization, ballooning degeneration, and confluent epidermal parakeratosis [1]. More commonly, however, histopathology is either non-specific, such as found in our patient, or displays upper epidermal pallor with psoriasiform hyperplasia and confluent parakeratosis [1, 11]. Diagnosis is made by clinical findings subsequently responsive to zinc supplementation supported by findings of low plasma or serum zinc concentration and/or suggestive histologicfindings [1, 11].

AIE is a rare cause of intractable diarrhea and malnutrition associated with gut autoantibodies and predisposition to autoimmunity [9, 12]. Histologically, there is partial or complete small bowel villous blunting, deep crypt lymphocytosis, increased crypt apoptosis, and minimal intraepithelial lymphocytosis. Diagnostic criteria necessitate chronic diarrhea (>6 weeks) with malabsorption refractory to dietary modification, presence of autoantibodies, no known immunodeficiency, and histologic findings that exclude other causes of villous atrophy [9]. Autoantibodies associated with AIE include antibodies against enterocytes, goblet cells, pancreatic islets, DNA, thyroglobulin, smooth muscle, and gastric parietal cell, the latter two of which were present in our patient [9].

Zinc, and other nutritional, deficiencies in adults are often a manifestation of an underlying malabsorptive etiology. With the rise of chronic diseases in adults, it has become increasingly difficult to determine the main cause of malabsorption in a patient with multiple chronic illnesses that individually predisposes to malnutrition. Clinical history and continuity of care become critical for establishing a clear timeline of symptoms onset and associations. Our patient had a 10-year history of Roux-en-Y gastric bypass with concurrent vegetarianism, pregnancy complicated by gestational hypertension and opioid dependence 2 years prior, and chronic hypertension treated with diuretics, each individual risk factors for zinc deficiency. However, zinc is not stored in large amount in the body [13], so given the chronicity of these medical issues and no history of acquired AE, they were unlikely to be the cause of her current presentation. The timing of hematochezia, sicca syndrome, and ITP closely following the onset of AE symptoms 4 months ago suggested that she likely developed AIE that presented initially with cutaneous findings of zinc deficiency secondary to gastrointestinal dysfunction.

Acquired AE has a very good prognosis with prompt intravenous supplementation starting at 3 mg/kg/day of elemental zinc. Recurrence is likely with untreated underlying conditions, so serum/plasma zinc levels and zinc-dependent enzyme levels should be monitored every 3 to 6 months. It is likewise advisable to monitor copper level and supplement if necessary since zinc can interfere with copper absorption [1, 14]. Reports have shown dramatic clinical improvements within the first few days to weeks of zinc supplementation, often ahead of normalization in serum zinc level [1, 3–7, 10], as illustrated in our patient.

## Conclusion

In summary, clinicians should maintain a low threshold of suspicion for acquired AE and check for zinc deficiency in adult patients with associated risk factors for malnutrition who presents with a confluence of relevant dermatologic findings that are refractory to standard therapy. Additionally, clinicians should also consider the possibility of a broader nutritional deficiency and an underlying primary malabsorption etiology. In investigating the latter for a patient with acquired AE, recognition of the association with adult AIE can benefit patient care by triggering earlier diagnosis, minimizing unnecessary tests, and establishing earlier interventions that can improve a patient's quality of life and prevent the recurrence of acquired AE.

## Abbreviations

AE: Acrodermatitis enteropathica
AIE: Autoimmune enteropathy
CPN: Central parenteral nutrition
IBD: Inflammatory bowel disease
ITP: Immune thrombocytopenic purpura
SJS: Stevens-Johnson Syndrome
TEN: Toxic epidermal necrolysis
